# Strategies to Prevent and Reduce Diabetes and Obesity in Sacramento, California: The African American Leadership Coalition and University of California, Davis

**DOI:** 10.5888/pcd10.130074

**Published:** 2013-11-14

**Authors:** Linda Ziegahn, Dennis Styne, Joyce Askia, Tina Roberts, Edward T. Lewis, Whitney Edwards

**Affiliations:** Author Affiliations: Dennis Styne, University of California Davis, Davis, California; Joyce Askia, Sacramento County Health and Human Services, Sacramento, California; Tina Roberts, Roberts Family Development Center, Sacramento, California; Edward T. Lewis, California Black Health Network, Inc, Sacramento, California; Whitney Edwards; California State University Sacramento, Sacramento, California.

## Abstract

**Background:**

Diabetes is one of the leading causes of illness and death for African Americans and people of African descent throughout the United States and in the city and county of Sacramento, California. The involvement of families and communities in developing prevention strategies can increase the likelihood that behavioral changes will be sustained.

**Context:**

Three member organizations of the African American Leadership Coalition (AALC) entered into a partnership with the University of California, Davis (UC Davis) to engage families in developing a process to identify barriers to diabetes and obesity prevention and reduction, exchange strategies, and create action plans for prevention.

**Methods:**

The intervention comprised 3 phases: 1) coalition formation and training; 2) data collection, analysis, and dissemination of results; and 3) development of family and community action plans. Academic and community partners planned and implemented all project phases together.

**Outcomes:**

Sources of information about diabetes and obesity were primarily doctors and the Internet; barriers were related to lack of time needed to prepare healthy meals, high food costs, transportation to fresh markets, motivation around healthy habits, and unsafe environments. Action plans addressed behavioral change and family cohesion. The group discussion format encouraged mutual support and suggestions for better eating and physical exercise habits.

**Interpretation:**

This collaborative partnership model can strengthen existing group relationships or promote new affiliations that form the basis for future action coalitions. Participants worked both within and across groups to exchange information, stories of success and challenges, and specific health improvement strategies.

## Background

In the National Health Interview Survey for January–September 2012, 9.2% of adults aged 18 or older had been given a diagnosis of diabetes, an increase from 5.1% in 1997: 13.1% for non-Hispanic blacks, 12.7% for Hispanics, and 7.1% for non-Hispanic whites (1). During this same period, 29.0% of adults in the United States aged 20 or older self-reported obesity: 41.7% of black women reported a BMI of 30 kg/m^2^ or more, as did 32.9% of black men, compared with 33.7% of Hispanic women and 31.3% of Hispanic men, and 26.3% of white women and 29.0% of white men (1).

African American young people are also at great risk for adult diabetes. According to the Centers for Disease Control and Prevention’s 2009 Youth Risk Behavior Survey, 21% of black students (23.3% of girls and 18.7% boys) are overweight, and an additional 15.1% of black students (12.6% girls and 17.5% boys) are obese (2). The National Health and Nutrition Examination Survey (NHANES) estimated that between the 1988–1994 survey and the 2009–2010 survey, the prevalence of obesity increased from 16.3% to 24.8% among black girls, and from 10.7% to 22.6% among black boys (3). Of 3,953 young people aged 3 to 19 with diabetes in the SEARCH for Diabetes in Youth final study sample, 429 had type 2 diabetes (31.0% African American, 21.2% Hispanic, and 19.1% white) (4).

Recent literature suggests that parental and community involvement is important for engaging children in physical activity (5,6). Unresolved family conflict and negative emotional tone in African American families can predict the course of type 2 diabetes (5). Similarly, greater physical activity and better self-assessed general health and diabetes management were found in families believing that life had order, meaning, and manageability. Education should involve not just family members with diabetes but the entire family (6). “Family cohesion,” how much adolescents feel understood and acknowledged by and enjoy being with their families, affects the influence of parents on adolescent physical activity (7). Functional and cohesive families are able to manage everyday tasks through supportive family interactions around management of physical activity and diet, shared decision making, appropriate boundaries, and effective conflict resolution strategies that support behavioral change (7,8).

For these reasons, the African American Leadership Coalition (AALC) of Sacramento decided to build on the family focus of its members. The AALC, which comprises more than 30 member organizations, wanted to learn more about connections between African American family dynamics and beliefs about prevention strategies and disease manageability and treatment in a community context. The University of California, Davis (UC Davis), which has long focused on type 2 diabetes and obesity in children and adults, joined with the AALC to submit an intermural grant proposal that was funded by a National Institutes of Health Clinical and Translational Science Award to UC Davis.

## Community Context

This exploratory study is a community–academic partnership to investigate how diabetes, obesity, and related health issues are perceived by families (primarily, but not exclusively African American) in Sacramento city and county. The African American population of the city of Sacramento is 14.6%, according to 2010 census data (9). Nearly 62% of all African Americans in Sacramento County are overweight or obese and 15% have received a diagnosis of diabetes (10). Only 20.7% of Sacramento teens were active 7 days a week in 2009, and 37% were active only 1 to 3 days per week (11). These statistics suggest that diabetes and obesity are significant problems in these communities and that prevention efforts could benefit from a family focus.

Many research projects highlight African Americans’ health problems from the perspective of community deficits, rather than assets (12). The AALC–UC Davis partnership wanted to build on community strengths. Therefore, 2 primary study objectives were identified. The first was to identify prior knowledge of a small sample of primarily African American families about diabetes and obesity, and to learn about their ideas on key prevention barriers and health improvement strategies. The second was to demonstrate that the synergy resulting from community dialogue among overlapping African American community groups, along with the experience of UC Davis researchers, could increase the effectiveness of prevention efforts for this population. Key features of this synergetic approach were adapted from the participatory research and coalition-building model developed by Giachello and colleagues (13).

## Methods

Our study used 3 distinct yet interconnected phases of collaborative data collection and analysis. The theory underlying this approach stemmed from the community-based participatory research (CBPR) model described by Minkler and Wallerstein (14) in which community members and their research partners jointly identify health priorities, appropriate modalities for data collection and analysis, and action plans. Similarly, the model of Giachello et al stressed capacity development and ongoing coalition formation from the beginning, building on community assets and targeted action so that new individual and family behaviors around diabetes prevention and management could be sustained (13). We condensed the 6 phases of the latter model into 3: 1) coalition formation and training; 2) data collection, analysis, and dissemination of results; and 3) development of family action plans (Table 1).

**Table 1:** Project Timeframe: African American Leadership Coalition and UC Davis, Sacramento, CA, October 2011–August 2012

Project Phase and Task Project Month	1	2	3	4	5	6	7	8	9	10
**Phase 1. Coalition formation/training**										
Proposal writing	x	x	x							
Sample selection	x	x	x							
IRB/CITI training		x	x							
CBPR methods training			x	x	x					
Final instrument design				x	x					
**Phase 2. Data collection, analysis, dissemination**										
Meeting 1: Focus groups: implementation, data analysis, presentation of results						x				
Meeting 2: Family survey: implementation/explanation, data analysis, presentation of results						x	x			
**Phase 3. Development of action **										
Meeting 3: Presentation of family survey results, explanation of family action plan survey								x		
Analysis of family action plans									x	
Meeting 4: Presentation of family action plan results, question and answer session: earlier participant questions, implementing new prevention strategies, next steps for project									x	x

### Phase 1. Coalition formation and training

In spring 2011, the Community Engagement Program of UC Davis’s CTSC organized a workshop on partnership formation, coupled with a call for proposals for community-engaged research grants. AALC members attending the workshop identified 3 member organizations with strong health interests: the Roberts Family Development Center (RFDC), the Christian Fellowship Ministry Church, and the local chapter of the National Council of Negro Women.

Study authors from these 3 organizations were viewed as leaders or possessed considerable knowledge of their group’s mission and operations. The resulting partnership application was funded. The project staff comprised 3 AALC community partners, a UC Davis pediatric endocrinologist serving as principal investigator, the CTSC community-engaged research specialist, and a student intern.

AALC community partners asked for 8 volunteers from each of their 3 organizations to represent families in the study. The total number of study participants was 24: 21 women and 3 men; 20 African Americans, 2 Latinas, and 2 Native Americans. Education levels ranged considerably: of the 20 participants who indicated highest level of education, 7 had post-baccalaureate training, 7 had attended some college or graduated, 5 had completed high school, and 1 had completed 10th grade. The average number of participants’ households was 2.76; 5 people lived alone, and 6 were members of a two-person household.

The first phase of coalition development lasted 5 months and included proposal writing and the eventual awarding of the grant. Additionally, all 6 project personnel were trained in research ethics through the Collaborative Institutional Training Initiative (CITI). The project was approved by the UC Davis Institutional Review Board (IRB).

The research specialist trained the 3 community partners and the intern in how to conduct focus groups, how focus group results can inform family health survey development, and how survey results can lead to the development of categories for family action plans.

### Phase 2. Data collection and analysis, and dissemination of results

This 2-month phase started with focus groups drawn from the church, the family development center, and the women’s group and was conducted in their corresponding community settings, where participants felt most at home. Focus group methodology has proven advantageous in exploring complex and sensitive topics such as diabetes and is consistent with the oral traditions of many cultures (6).

The community partner for each group served as focus group facilitator, and the intern and research specialist took notes for all 3 meetings. Community partners were selected as facilitators because we wanted to capitalize on the history and trust that each partner had within his or her group. The community partners felt strongly that bringing in unknown facilitators would not instill the trust necessary for candid responses. Training in the neutral framing of focus group questions helped facilitators conduct sessions in an unbiased manner. Facilitators asked participants to describe the importance of diabetes and obesity in their families and communities, sources of information about these diseases, barriers to getting healthful eating and exercise resources, and to answer specific questions that should be asked in the second family survey study phase.

All 6 project personnel, with guidance from the research specialist, reviewed transcriptions of focus group notes. We identified the following key topics through qualitative analysis (15): barriers to better diet and exercise (eg, motivation and denial, work demands, fast food advertising, not enough exercise programs for children, cost of fresh produce, transportation to good grocery stores), family perceptions of obesity and diabetes, and information sources on prevention (television, Internet, doctors, schools). Here are a few typical responses: “When my husband had diabetes, I had to educate myself – through the Internet, books, hospital classes, and Isaac Hayes’s cookbook.” “Mom eats fast food in her room, kids in theirs. How do you make [healthy eating] cool?” “It’s so much easier when you got folks around to keep you going.”

Focus group analysis led to construction of an 18-item family survey (Appendix A) that measured health and support around diabetes (16,17). At the second group meetings, focus group themes were presented and facilitators explained how these themes informed survey development and clarified each question. No further survey adjustments were made, and participants were asked to complete the survey with the help of their families. 

Twenty-one of 24 surveys were returned. Again, the project team tabulated responses and identified key themes, which led to development of the family action plan grid. Throughout the second phase, the project team met once or twice a month to review data from focus group and family survey instruments and plan for Phase 3 activities.

### Phase 3. Development of action plans — family and community

At the third meeting of each group, we presented a slideshow summarizing data from family surveys of all 3 groups and how survey results informed family action plan grids. We then asked participants to complete these grids with their families. Participants could list up to 5 specific goals around diabetes and obesity prevention, family members involved, definitions of success for each goal, starting dates, and check-in dates to monitor progress. Once action plans were returned, the project team again tabulated results.

For the fourth and final meeting, all 3 groups came together at the Roberts Family Development Center. At this point, participants were comfortable with sharing personal stories about challenges of combatting obesity and diabetes-related diseases and potential solutions. The trust that had existed within group was extended across groups, as evidenced by the ease and enthusiasm participants expressed as they shared their completed action plans. Next, the UC Davis pediatric endocrinologist led a discussion focused on the specific questions participants raised in the family surveys as well as more general questions. The meeting closed with a discussion about how participants planned to continue working on goals — as individuals, families, and communities.

Although there were a total of 24 study participants, attendance varied at meetings throughout the 3 phases. Most participants attended all 4 meetings, but usually 1 or 2 participants were missing at each meeting. Gift card compensation was provided for attendance at each meeting and for completion of the study.

## Outcomes

Key findings for this project came from data collection instruments and from observing interactions of participants with one another and with the project team during the 4 community meetings for each group (Appendix B provides results for each of the family survey questions asked in phase 2). We asked participants if they themselves had diabetes or felt they were obese, and whether members of their immediate family had these same problems. Of 21 participants completing the family survey, 3 reported having diabetes, and 6 described themselves as obese. Although only 3 participants claimed partners or children with these diseases, 11 described parents or other close relatives as having diabetes, and 7 described these same people as obese. Perhaps most informative, participants shared stories during group meetings of families struggling for generations with “sugar,” of motivational challenges to losing weight, and of how dietary habits had changed over generations for the worse. Participants spoke of disappointments and successes, both within their community group meetings and at the fourth meeting with all 3 groups present.

Participants enumerated the health problems accompanying diabetes and obesity (high blood pressure, heart disease, high cholesterol, and death). When asked in the survey to mention foods considered healthy they listed fruits, vegetables, whole grains, and lean meats — all standard components of the USDA diet guidelines (18). Walking was the most common physical activity reported, ranging from 10 reporting 30–60 minutes per week to 6 reporting more than 3 hours weekly.

Participants had questions about the causes and effects of diabetes and obesity, about prevention and cures, and about the best ways to motivate their families — and themselves — to stay healthy (Table 2). The biggest sources of information were doctors (17 responses), the Internet (15), books and newsletters (9), family and friends (9), television (6), churches (6), and schools (3).

**Table 2:** Participants’ Questions about Diabetes and Obesity: African American Leadership Coalition and UC Davis, Sacramento, California, October 2011–August 2012

**What would you like to know about diabetes or obesity that you don’t already know?**
**Causes and effects**
What medical conditions, or genetics, cause diabetes?
Why does diabetes result from obesity or overweight?
What are the current standards to determine obesity and have they changed?
What are the effects of medications for diabetes and obesity?
**Prevention and cures**
How can diabetes be prevented?
Can medicine cure diabetes?
Can holistic care cure diabetes?
Can diabetes and obesity be controlled *without* medication?
How can a person older than 70 years reverse diabetes through exercise?
**Motivation**
How can we make losing weight easy and fun?
How can we motivate our families?

Participants in all 3 groups identified similar barriers to healthful eating and exercise in focus groups and family surveys: lack of knowledge, transportation to stores and markets, motivation, miscommunication with doctors about healthful eating, unsafe neighborhoods making exercise difficult, not having enough time to cook, no physical education in schools, and need for more education on diet and exercise for young people.

Although participants were knowledgeable about healthful diets and the effects of unchecked diabetes and obesity, the barriers mentioned made it difficult to act consistently. A strength of the AALC–UCD project was the opportunity for mutual support that resulted from regular group meetings. In the fourth and final meeting, a young woman asked the others how they managed to cook healthy meals for children when working late and having to drive children to activities. The women and men in the group shared strategies: “batch” cooking at the beginning of the week, having healthful snacks in the car, and looking for healthier fast-food options. As participants got to know one another in the 4 meetings, the voices heard in each session changed; participants who said little initially felt increasingly comfortable asking questions and sharing potential diabetes and obesity reduction strategies.

Results of the family action plans revealed participants’ interests in change at both the individual and collective levels (Table 3). Most of the goals focused on improving eating habits and increasing physical activity. However, preventing diabetes and obesity was also viewed in terms of family cohesion — developing stronger relationships and embracing a more holistic life style that reflected not only better health habits and greater emphasis on taking care of oneself, but also greater balance between family, work, and spiritual life.

**Table 3:** Results: Family Action Plans of African American Family Representatives, African American Leadership Coalition and University of California, Davis, Sacramento, California, October 2011–August 2012

Goals	No. of Responses^a^	Examples	Measures of Success
Eat healthier, live healthier	29	More fruit, vegetables, food from garden Stop eating high-calorie snacks, soda Cook at home: batch meals for week, instead of eating out Drink 8 glasses of water a day Stop eating red meat; keep food diary; lower sugar intake	Weight loss Buy less fatty foods Make meal decisions as family Watch “Supersize Me” video Family will get along better
Physical activity	21	Walk along river with family, on treadmill 35–45 min/d; 15–20 min/d with husband Work out: plan for me and son, join gym, increase muscles, decrease body fat 20%, aerobics Outside play for kids Swimming, dancing, running, improving cardio	Weight loss Better stamina Toning, building muscle Plan schedule of activities
Educate family	10	Education about obesity, sugary drinks, snacks Read about new health issue each month Educate congregation (from pastor) Tell others what I’ve learned about diabetes	Dine out less Make meals special Raise kids’ awareness about family health history Develop healthy relationships with children
Deal with diabetes	7	Get tested Increase knowledge, teach others Care better for family members with diabetes	Make sure we don’t have diabetes Outreach to all kinds of people Control over condition
Motivation	6	Motivate family around diabetes prevention, healthful foods, and exercise Motivate children around plan for exercise Encourage others to exercise, encourage congregation around diabetes and obesity prevention	More frequent discussion around prevention strategies More outdoor sports More family time, trust, love Spreading prevention message
Doctors’ appointments	4	Regular check-ups around diabetes testing Keep doctors’ appointments Ask questions during visits	Living well, spiritually and emotionally No prescriptions or medical issues
Other	8	Environmental factors: retire from job to more fun, healthful activities Move to a cheaper house Keep taking iron pills for better red cell count Stop smoking Meditate 20 min/d Take less medication	Prevent heart disease, diabetes Change eating habits after losing weight

a Based on number of responses falling into each goal category. Goal categories were constructed from content analysis of action plans.

The community partners, valued as leaders by the 3 groups, engendered trust for future coalitions by building on existing intergroup relations and instilling confidence and community self-reliance across the 3 groups. Equally important, community partners and UC Davis project staff learned from participants. At the last all-group meeting, the pediatric endocrinologist spoke of how encouraged he was to hear creative approaches to losing weight, such as exercising as a family to gospel music, and starting walking programs that led to weight loss and improved conditioning. In addition, older parents supported younger parents who struggled to find time to cook healthful meals for their children.

Finally, the AALC-UC Davis project team learned about administrative and research procedures associated with federal grants. For instance, they discovered that the UC Davis contract specialists had little experience in setting up contracts between the university and community organizations. Also, community partners found the IRB approval process longer than expected. On the other hand, the research ethics training all project personnel completed was valued by community partners because they became more aware of the thorough approval process for academic research projects and the ethical considerations associated with human subjects research.

## Interpretation

This project succeeded in providing data on perceived barriers and motivators around preventing diabetes and obesity among a small but representative group of diverse African Americans in Sacramento. It also provided a framework for how groups might work together in the future. For example, participants expressed interest in focusing future mutual support interventions on faith-based communities, given the solidarity among members and the preeminent role of pastors. Academic-community partnerships often train lay leaders as health educators and use such culturally acceptable activities as praise aerobics, chair exercises set to gospel music, and inclusion of scripture related to weekly health topics (19).

In summary, the 3 community groups were consistent about the difficulties faced by families in trying to keep themselves and their families healthy. Social pressures that limit the amount of time spent with family; a paucity of nutritious, affordable food; and unsafe neighborhoods made acting on the knowledge of what constitutes a healthful diet and adequate exercise challenging. At the same time, the groups consistently expressed enthusiasm for learning more and harnessing the power of group and community interaction to follow through with plans around specific behavioral changes. In addition, several people became more outspoken in seeking answers to their health questions in public. All wanted the project to continue.

Follow-up research projects will explore how UC Davis can leverage prevention resources and activities with the 3 AALC community organizations in this study, other organizations serving the needs of underserved African American populations, and faith-based institutions.

The process of identifying issues and prevention barriers, investigating family health status around diabetes and obesity, and developing actions plans for behavioral and community change can be replicated and disseminated. Critical to the sustainability of such coalition-building efforts is building on the dynamism of small community groups who know and trust their members and leaders, along with shared, transparent project administration by both community and academic partners. Just as critical is the commitment by partnership leaders to follow up this initial problem identification, data gathering, and action planning with health resources and activities to sustain behavioral and environmental change at the individual, family, and community levels.

## Appendices

### Appendix A. Community Participation in Improving Health Status around Diabetes and Obesity: Survey Instrument.

This file is available for download as a Word document.

### Appendix B. Community Participation in Improving Health Status around Diabetes and Obesity: Results.

This file is available for download as a Word document.
