# Supplementary material for: Strategies to Prevent and Reduce Diabetes and Obesity in Sacramento, California: The African American Leadership Coalition and University of California, Davis
Source: Prev Chronic Dis. 2013 Nov 14;10:E187. doi: 10.5888/pcd10.130074 (PMC3830925; doi:10.5888/pcd10.130074)
Supplement: Supplementary file 1 [file 13_0074_01.doc]

Appendix A.

Community Participation in Improving Health Status around Diabetes and Obesity

**Family Survey Instrument**

Welcome!

We appreciate your participation in this important study, which is a partnership between the University of California Davis and the African American Leadership Coalition.

The focus of this survey—the second phase of our study—is on learning more about your family’s experiences with health, particularly around issues connected to diabetes and obesity. **Please note that sometimes questions in the survey are for you personally, and sometimes we are asking you about health habits and attitudes of your family members.**

When you have completed this study, please mail it by **June 15** to your study representative in the stamped envelope provided with this survey.

Thank you very much for your time!

Tina Roberts Dennis Styne, MD

Roberts Family Development Center UC Davis Dept. of Pediatrics

Co-Principle Investigator Principle Investigator

Community Participation in Improving Health Status around Diabetes and Obesity

**Family Survey Instrument**

**Demographic data:**

1. Ethnic origin

_____Black, African/African American/Afro-Caribbean but non-Hispanic

_____Hispanic

_____White, non-Hispanic

_____Filipino

_____Asian or Pacific Islander

_____American Indian/Alaskan Native

_____Other______________________

1. Gender

_____Male

_____Female

1. Please **circle** the highest year of school you have completed:

1 2 3 4 5 6 7 8 9 10 11 12 13 14 15 16 17+

Primary High School College Post-college

1. What is the age range for each person in your family living with you? (**Please check one age box for each family member**)

**Do NOT write any names of family members on this table.**

| PERSON | AGE  infant-5 | 6-10 | 10-14 | 15-19 | 20--29 | 30-39 | 40-49 | 50-59 | 60-69 | 70-79 | 80+ |
| --- | --- | --- | --- | --- | --- | --- | --- | --- | --- | --- | --- |
| **Self** |  |  |  |  |  |  |  |  |  |  |  |
| Spouse/partner |  |  |  |  |  |  |  |  |  |  |  |
| Child |  |  |  |  |  |  |  |  |  |  |  |
| Child |  |  |  |  |  |  |  |  |  |  |  |
| Child |  |  |  |  |  |  |  |  |  |  |  |
| Child |  |  |  |  |  |  |  |  |  |  |  |
| Mother |  |  |  |  |  |  |  |  |  |  |  |
| Father |  |  |  |  |  |  |  |  |  |  |  |
| Other family |  |  |  |  |  |  |  |  |  |  |  |

**HEALTH STATUS**

1. In general, how would YOU rate your current health or well-being? **(Circle one)**

1 2 3 4 5

Excellent Very good Good Fair Poor

1. Have you or anyone in your immediate family been diagnosed with diabetes and/or obesity?

**(Check all that apply)**

SELF ____Yes ____No **IF YES:** ___diabetes ___obesity

Spouse/partner ____Yes ____No ___diabetes ___obesity

Child/children ____Yes ____No ___diabetes ___obesity

Your mother ____Yes ____No ___diabetes ___obesity

Your father ____Yes ____No ___diabetes ___obesity

Other close relative ____Yes ____No ___diabetes ___obesity

1. Where do you typically get your information on diabetes or obesity? **(Check all that apply)**

____Doctors

____Internet

____Books or newsletters on health

____Family members or friends

____Television

____Faith-based organizations (e.g., church, temple, mosque, etc.)

____Schools

____Other (please list: ­­­­______________________________________________)

1. Please rate your knowledge about diabetes on a scale of 1 – 5 (**Circle one**):

1 2 3 4 5

Very low Low Moderate High Very high

9 a. Please rate your knowledge about obesity on a scale of 1 – 5 (**Circle one**):

1 2 3 4 5

Very low Low Moderate High Very high

9 b. What would you like to know about diabetes or obesity that you do not already know?

________________________________________________________________________

________________________________________________________________________

________________________________________________________________________

________________________________________________________________________

________________________________________________________________________

1. What are barriers for you and family members to getting better care around diabetes and obesity?

**(Check all that apply)**

____Lack of insurance

____Communication with doctors

____Lack of knowledge on resources around these diseases

____Lack of transportation to doctor/health facility

____Other (please list: ­­­­______________________________________________)

11. Do you consider yourself overweight or obese?

____Yes

____No

12. Has your doctor informed you that you are overweight or obese?

____Yes

____No

13 a. Is anyone in your immediate family overweight or obese?

____Yes

____No

If yes, who? **(Check all that apply)**

____Mother

____Father

____Spouse
 ____Child/children

____Other (please list: _____________________________________________)

13 b. Do you think there are risks to being overweight or obese? **(Check one)**

____Yes

____No

____Not sure

13 c. If yes, what are some of the risks?

_______________________________________________________________________________

_______________________________________________________________________________

_______________________________________________________________________________

_______________________________________________________________________________

_______________________________________________________________________________

14 a. List three foods that you think should be in a healthy meal:

1. _____________________________________________
2. _____________________________________________
3. _____________________________________________

14 b. How often do you and your family eat healthy meals? (**Circle one**)

1 2 3 4 5

Never Rarely Sometimes Almost Always

Always

14 c. Please check any of the following that make it challenging to eat healthy or healthier:

**(Check all that apply)**

____Lack of knowledge about what foods are considered healthy

____Lack of nearby grocery stores or markets with healthy foods

____Lack of transportation to sources of healthy foods

____The high cost of healthy foods

____Lack of time needed to prepare healthy meals

____Other (please list: __________________________­­____________________

_____________________________________________________________)

15 a. How often do YOU exercise? (**Circle one**)

1 2 3 4 5

Never Rarely Sometimes Almost Always

Always

15 b. What kinds of physical activity do YOU engage in? Please check the box that shows the **amount of time**

you spend doing any of the following activities:

| ACTIVITY | No time | Less than 30  minutes per week | 30-60 minutes  per week | 1-3 hours per week | More than 3 hours per week |
| --- | --- | --- | --- | --- | --- |
| Stretching, strengthening |  |  |  |  |  |
| Walking |  |  |  |  |  |
| Swimming |  |  |  |  |  |
| Bicycling |  |  |  |  |  |
| Aerobic exercise |  |  |  |  |  |
| Running |  |  |  |  |  |
| OTHER (write in  below) |  |  |  |  |  |

16. Please check any of the following that make it difficult or challenging for you to exercise:

**(Check all that apply)**

____No place to walk

____Unsafe environment for outside activity

____No access to equipment

­____No time for exercise

____Not interested

____Health problems (please list:______________________________________)

____Other (please list:_______________________________________________

_____________________________________________________________)

17 a. Do you feel other members of YOUR FAMILY get enough exercise? **(Check one)**

____Yes

____No

17 b. Please check any of the following that make it difficult or challenging for YOUR FAMILY MEMBERS to

exercise: **(Check all that apply)**

____No place to walk

____Unsafe environment for outside activity

____No access to equipment

­____No time for exercise

____No physical education program in schools

____Not interested

____Health problems (please list:______________________________________)

____Other (please list:_______________________________________________

_____________________________________________________________)

18. Where do you and your family members receive medical care? **(Check all that apply)**

____family physician

____community clinic

____emergency room

____I do not receive medical care

____Other (please list:_______________________________________________)

**THANK YOU VERY MUCH!**
