# Supplementary material for: Strategies to Prevent and Reduce Diabetes and Obesity in Sacramento, California: The African American Leadership Coalition and University of California, Davis
Source: Prev Chronic Dis. 2013 Nov 14;10:E187. doi: 10.5888/pcd10.130074 (PMC3830925; doi:10.5888/pcd10.130074)
Supplement: Supplementary file 2 [file 13_0074_02.doc]

Appendix B.

Community Participation in Improving Health Status around Diabetes and Obesity

**Family Survey Instrument**

Welcome!

We appreciate your participation in this important study, which is a partnership between the University of California Davis and the African American Leadership Coalition.

The focus of this survey—the second phase of our study—is on learning more about your family’s experiences with health, particularly around issues connected to diabetes and obesity. **Please note that sometimes questions in the survey are for you personally, and sometimes we are asking you about health habits and attitudes of your family members.**

When you have completed this study, please mail it by **June 15** to your study representative in the stamped envelope provided with this survey.

Thank you very much for your time!

Tina Roberts Dennis Styne, MD

Roberts Family Development Center UC Davis Dept. of Pediatrics

Co-Principle Investigator Principle Investigator

Community Participation in Improving Health Status around Diabetes and Obesity

**Family Survey Instrument**

(with results)

**Demographic data:**

1. **Ethnic origin**

|  | N=24* |
| --- | --- |
| Black non-Hispanic | 20 |
| Hispanic | 2 |
| Asian or Pacific Islander | 2 |

1. **Gender**

|  | N=24* |
| --- | --- |
| Male | 3 |
| Female | 21 |

1. **Highest year of school completed**

|  | N=19 |
| --- | --- |
| 10^th^ grade | 1 |
| High School | 4 |
| College (1 year or graduated) | 7 |
| Post-College | 7 |

1. **Age Range of Family Members in Household**

| PERSON | AGE  infant-5 | 6-10 | 10-14 | 15-19 | 20--29 | 30-39 | 40-49 | 50-59 | 60-69 | 70-79 | 80+ |
| --- | --- | --- | --- | --- | --- | --- | --- | --- | --- | --- | --- |
| **Self** |  |  |  |  | 1 | 3 | 4 | 5 | 6 |  | 1 |
| Spouse/partner |  |  |  |  | 1 | 2 | 1 | 1 | 2 |  |  |
| Child | 3 | 6 | 2 | 1 |  |  | 1 |  |  |  |  |
| Child | 2 | 2 | 4 |  |  |  |  |  |  |  |  |
| Child | 1 |  | 1 | 1 |  |  |  |  |  |  |  |
| Child |  |  |  |  |  |  |  |  |  |  |  |
| Mother |  |  |  |  |  |  |  | 2 |  |  | 1 |
| Father |  |  |  |  |  |  |  | 1 |  |  |  |
| Other family |  |  |  |  | 1 |  |  |  |  |  |  |

**HEALTH STATUS**

1. **Rating of current health or well-being**

|  | N=20 |
| --- | --- |
| Excellent | 2 |
| Very good | 3 |
| Good | 10 |
| Fair | 4 |
| Poor | 1 |

1. **Family members diagnosed with diabetes or obesity**

|  | Diabetes | Obesity |
| --- | --- | --- |
| Self | 3 | 6 |
| Spouse/Partner | 1 | 1 |
| Child/children | 0 | 2 |
| Mother | 4 | 1 |
| Father | 1 | 1 |
| Other close relative | 6 | 5 |

1. **Source of information on diabetes or obesity**  **(Check all that apply)**

| Doctors | 17 |
| --- | --- |
| Internet | 15 |
| Books or health newsletters | 9 |
| Family members or friends | 9 |
| Television | 6 |
| Faith-based organizations | 6 |
| Schools | 3 |
| Other | 4 |

1. **Rating of knowledge about diabetes**

|  | N=20 |
| --- | --- |
| Very high | 5 |
| High | 3 |
| Moderate | 10 |
| Low | 1 |
| Very low | 1 |

9 a. **Rating of knowledge about obesity**

|  | N=20 |
| --- | --- |
| Very high | 5 |
| High | 6 |
| Moderate | 6 |
| Low | 2 |
| Very low | 1 |

9 b. **Other learning interests around diabetes and obesity**

- No answer-6
- Activities that make losing weight easy and fun
- What is a good motivator for family who doesn’t know the risk of either.
- Diabetes- different levels and how threatening each can be
- Obesity- how is the difference of being overweight and obesity determined in children under 12?
- What medical condition can cause obesity? - Like ‘genes’
- Why does diabetes come when you are obese or overweight?
- Does eating about a pound of candy per day cause diabetes?
- Cause of diabetes
- How to control diabetes and obesity without medication
- What are the current standards to determine obesity and has it changed?
- How to prevent it
- How a person reverses diabetes through exercise at the age of 70
- Know more about the effects of media med perception for diabetes/obesity (pros and cons, lack of issues, obesity, diabetes)
- Different ways to manage and control it, also if it can be done in different languages
- Ca it be cured with medicine?
- Can it be cured with holistic care? ( not maintained, cured)

1. **Barriers to getting better care around diabetes and obesity**

- None
- Lack of health care
- Not wanting to talk or deal with it
- Individual concern about health
- Lack of access to healthy and natural fruits and vegetables
- Transportation
- Communication with each other
- Communication with physicians and HMO
- Daycare
- Time
- Holistic
- Insurance

**11. Do you consider yourself overweight or obese?**

|  | N=18 |
| --- | --- |
| Yes | 11 |
| No | 7 |

**12. Has your doctor informed you that you are overweight or obese?**

|  | N=17 |
| --- | --- |
| Yes | 12 |
| No | 5 |

13 a. Is anyone in your immediate family overweight or obese?

|  | N=20 |
| --- | --- |
| Yes | 15 |
| No | 5 |

If yes, who? **(Check all that apply)**

| Mother | 7 |
| --- | --- |
| Father | 1 |
| Spouse | 3 |
| Child/children | 5 |
| Other | 9 |

13 b. **Do you think there are risks to being overweight or obese?** Yes = 20

13 c. **If yes, what are some of the risks?**

| Health problems | 16 |
| --- | --- |
| Death | 6 |
| Bad food choices | 2 |
| Longevity, self-esteem, high blood pressure,  diabetes, heart disease, high cholesterol |  |

**14 a. Three foods that should be in a healthy meal**

| Vegetables | 21 |
| --- | --- |
| Poultry | 12 |
| Grain | 9 |
| Protein | 2 |
| Other: brown rice, olive oil, white bread, low sodium,  fiber, beans |  |

**14 b. Frequency of eating healthy meals**

|  | N=19 |
| --- | --- |
| Always | 1 |
| Almost always | 7 |
| Sometimes | 10 |
| Rarely | 1 |
| Never | 0 |

14 c. **Challenges to eating healthy or healthier**

**(Check all that apply)**

| Lack of time needed to prepare healthy meals | 13 |
| --- | --- |
| High cost of healthy foods | 11 |
| Lack of nearby grocery stores, markets w/healthy foods | 8 |
| Lack of knowledge—foods considered health | 5 |
| Lack of transportation-sources of healthy foods | 2 |
| Other: lack of job, don’t like the taste, lack of education ,  unwilling to cook healthy foods |  |

15 a. **How often do YOU exercise?**

|  | N=19 |
| --- | --- |
| Always | 1 |
| Almost always | 7 |
| Sometimes | 10 |
| Rarely | 1 |
| Never | 0 |

**15 b. Kinds of physical activities you engage in and time:**

| ACTIVITY | No time | Less than 30  minutes per week | 30-60 minutes  per week | 1-3 hours per week | More than 3 hours per week |
| --- | --- | --- | --- | --- | --- |
| Stretching, strengthening | 2 | 6 | 8 | 2 |  |
| Walking |  | 1 | 10 | 4 | 6 |
| Swimming | 9 | 1 | 2 |  |  |
| Bicycling | 8 | 3 | 1 |  |  |
| Aerobic exercise | 7 | 2 | 3 |  | 1 |
| Running | 7 |  | 1 |  |  |
| OTHER (write in  below) | 1 |  |  |  |  |
| dancing |  |  |  | 2 | 1 |
| Yoga |  |  | 1 |  |  |
| Push up |  |  | 1 |  |  |
| Skating |  |  | 1 |  |  |
| House cleaning |  |  | 6 |  | 1 |
| Gardening |  |  |  | 1 |  |

16. **Please** **check any of the following that make it difficult or challenging for you to exercise:**

**(Check all that apply)**

| Health problems | 7 |
| --- | --- |
| Unsafe environment-outside activity | 5 |
| No time for exercise | 5 |
| No access to equipment | 4 |
| Not interested | 4 |
| No place to walk | 2 |

**17 a. Do you feel other members of YOUR FAMILY get enough exercise? (Check one)**

|  | N=17 |
| --- | --- |
| Yes | 5 |
| No | 12 |

17 b. **Please check any of the following that make it difficult or challenging for YOUR FAMILY MEMBERS to**

**exercise: (Check all that apply)**

| Not interested | 7 |
| --- | --- |
| No access to equipment | 6 |
| No time for exercise | 6 |
| No physical education programs in school | 5 |
| Not interested | 4 |
| No place to walk | 1 |

**18. Where do you and your family members receive medical care? (Check all that apply)**

|  | N=21 |
| --- | --- |
| Family physician | 16 |
| Community clinic | 3 |
| I don’t receive medical care | 2 |

**THANK YOU VERY MUCH!**

*Data on gender and ethnicity came from original information from community partners, not survey.
